# Microbiota of Chronic Diabetic Wounds: Ecology, Impact, and Potential for Innovative Treatment Strategies

**DOI:** 10.3389/fmicb.2017.01791

**Published:** 2017-09-21

**Authors:** Sónia G. Pereira, João Moura, Eugénia Carvalho, Nuno Empadinhas

**Affiliations:** ^1^Center for Neuroscience and Cell Biology, University of Coimbra Coimbra, Portugal; ^2^Polytechnic Institute of Leiria Leiria, Portugal; ^3^Department of Geriatrics, University of Arkansas for Medical Sciences, Little Rock AR, United States; ^4^Arkansas Children’s Hospital Research Institute, Little Rock AR, United States

**Keywords:** microbiome, microbiota and immunity, diabetic foot ulcers, biofilms, novel therapeutics, infection, immune response, diabetes complications

## Abstract

World Health Organization considered diabetes as one of the 20th century epidemics, estimating that over 10% of the world population is diabetic or at high risk. Self-assessment studies indicate that diabetic patients consider chronic wounds to affect their quality of life more dramatically than vision loss or renal failure. In addition to being the main reason for diabetic patients’ hospitalization, the economic burden of diabetic chronic wounds is close to 1% of United Kingdom and United States health systems budgets, which exceeds the funds allocated to the treatment of some types of cancer in both countries. Among the factors preceding the emergence of chronic diabetic wounds, also designated diabetic foot ulcers (DFUs), hygiene and pressure in specific areas are under patient control, while others are still far from being understood. A triple impairment in the innervation, immune responses, and vascularization associated to DFU has been extensively studied by the scientific community. However, the skin natural microbiota has only recently emerged as having a tremendous impact on DFU emergence and evolution to chronicity. Despite the great inter- and intra-variability of microbial colonizers, ongoing efforts are now focused on deciphering the impact of commensal and pathogenic microbiota on DFU etiology, as well as the mechanisms of interkingdom microbial–host communication. This review summarizes recent work in this context and offers new microbiological perspectives that may hold potential in the prevention and treatment of chronic diabetic wounds.

## Introduction

Diabetes is the most prevalent endocrine disease worldwide, considered by the World Health Organization as one of the 20th century epidemics (Wild et al., 2004). Almost 10% of global adult population is diabetic or at risk of developing diabetes (World Health Organization [WHO], 2016). Severe secondary and highly impairing pathologies are associated to this disease. Some of the pathologies diabetic patients often face include kidney failure requiring dialysis or transplantation, vascular disease with high risk for heart failure and stroke, and blindness. Chronic wounds are also among the most devastating conditions in these patients, caused by an impaired immune response and an associated high microbial burden that frequently leads to amputation, mainly of the lower limbs (Wild et al., 2004; Icks et al., 2011; Forlee, 2011; Kerr, 2012; Barshes et al., 2013; World Health Organization [WHO], 2016).

**FIGURE 1:**
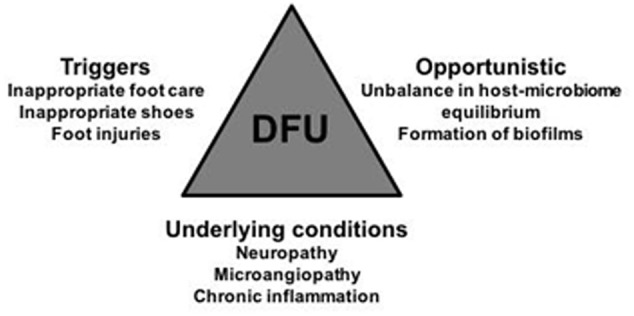
Visual representation of the major stakeholders that have an impact in DFUs emergence, evolution, and chronicity maintenance: the triggers, under patients control, the underlying conditions associated to diabetes; and the opportunistic microbes qualitative and quantitative shifts. Their combined influence in DFU is multifactorial and time-dependent, which further hinders the implementation of appropriate DFU therapies. Physicians, and scientists, should always keep in mind this trilogy when deciding their strategies.

Skin lesions are common to every individual during their lifespan with little or no care required, but for diabetic patients every tiny scratch can be a matter of great concern. Every small lesion can become a seriously infected chronic wound, ultimately leading to amputation or even death (Swartz, 2004). This is a consequence of diabetic-related impairments that thwart an adequate immune response against pathogens (Ming et al., 2012; Mirza et al., 2014; Moura et al., 2014; Leal et al., 2015). The skin microbiota of diabetic patients is also a contributing factor (Ki and Rotstein, 2008; Gontcharova et al., 2010; Martin et al., 2010; Foxman and Rosenthal, 2013; Gardner et al., 2013; Redel et al., 2013; Spichler et al., 2015; **Figure 1**). There has been a long time awareness that the human body has a natural microbiota, important for many physiological functions but also potentially pernicious if disturbed. Its composition, diversity, and distribution, as well as its dynamic interactions with the host were, until very recently, completely enigmatic. Recent advances in molecular technologies allowed the simultaneous sequencing of large amounts of different DNA (high-throughput DNA sequencing). This technical progression deemed us the possibility to finally start to understand which are those unknown microbial players in our body, with the human microbiome (the genetic fingerprints of our microbiota) project as the most ambitious endeavor so far (Human Microbiome Project Consortium [HMPC], 2012). Apart from this extensive study focused on healthy individuals, microbiologists took advantage of these new tools to seek relevant comprehensive information about infectious diseases. In this context, several studies emerged in the last few years, and the diabetic chronic wounds were not an exception (Gontcharova et al., 2010; Martin et al., 2010; Kong and Segre, 2012; Oates et al., 2012; Foxman and Rosenthal, 2013; Gardner et al., 2013; Redel et al., 2013; Spichler et al., 2015).

## Epidemiology, Social, and Economic Burden of Diabetic Chronic Wounds

**FIGURE 2:**
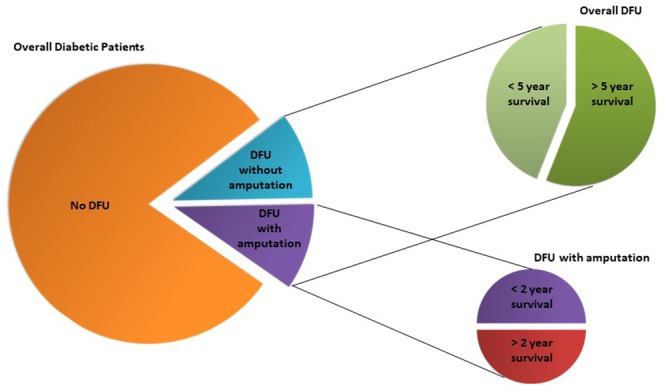
Diabetic foot ulcers (DFU) incidence in diabetic population and respective overall life expectancies according to DFU treatment (amputation vs. no amputation). Although luckily the majority of diabetic patients are not affected by DFU, those that have the unfortunate of developing it, have high probability of a short-term death sentence of 2 or 5 years, which is urgent to circumvent.

Chronic wounds are one of the most serious health problems related to diabetes. About 15–20% of the 420 million diabetic patients (World Health Organization [WHO], 2016) develop skin wounds across their lifespan, the majority evolving toward chronicity, particularly in the lower limbs. They are usually described as diabetic foot ulcers (DFUs) and are very difficult to treat, and hospitalization is often required to implement direct therapeutic procedures and patient care. In fact, and quite surprisingly, DFU are the most frequent upstream cause of hospitalization in diabetic patients (Forlee, 2011). Regardless all the therapeutic approaches, a huge number of diabetic chronic wounds degenerate to more severe stages of chronicity, with amputation of the affected limb as the lattermost resource for more than half of DFU patients (Icks et al., 2011; Kerr, 2012), when invasion and necrosis of the soft tissues and bones occur. Even still, this procedure frequently fails, leading to further amputations and ultimately to death. Indeed, on average amputation only increases life expectancy up to 2 years in half of the diabetic patients undergoing the procedure (Icks et al., 2011; Kerr, 2012). Regardless of this invasive intervention, it is estimated that only around 56% of diabetics with ulcerative wounds survive more than 5 years after their initial manifestation (Kerr, 2012; **Figure 2**).

Self-assessment studies report that diabetic patients consider DFU and the associated amputation to impact their life quality more dramatically than renal failure or sight loss, with depression and anxiety highly associated to the loss of a limb (Martin et al., 2010; Barshes et al., 2013). Several reports consider the loss in life quality of diabetic patients and their families to be higher than that of cancer patients (Kerr, 2012). Apart from the dramatic impairment DFU and amputation represent to the patient, such limitation also greatly affects the patient’s family wellbeing, by lowering the income and significantly increasing health costs (Icks et al., 2011; Kerr, 2012). For the national health systems, the direct and indirect costs associated to this pathology are also quite impressive. Around 17 billion dollars are annually spent in the United States for diabetic foot care, which is more than the funds allocated to the treatment of breast cancer and other types of cancer (Barshes et al., 2013). In the United Kingdom 0.7% of the total health costs, around 650 million pounds, are spent on DFU management (Kerr, 2012) and in developing countries, lower but still significant values, are also used by debilitated healthcare systems to deal with this pathology (Cavanagh et al., 2012).

Strategies to improve current therapy outcomes and, more importantly, to prevent wound emergence are imperative, not only for the sake of patients’ lives and their families, but also of healthcare systems economies.

## Etiology of Diabetic Chronic Wounds and A Quest for Skin Microbiota Signatures

There are several factors associated to the emergence of diabetic chronic wounds, with some under the patients’ control. Apart from a poor glycemic control that progressively leads to peripheral neuropathies, poor hygiene habits, lack of regular surveillance of skin integrity, and premature detection of lesions as well as prolonged pressure in specific areas, are known to collectively favor the emergence and progression of wounds, particularly in the foot. If properly controlled, these factors could in fact avert wounding or wound progression (Forlee, 2011). Physiological factors, not under the direct control of the patient, are also frequently involved in wound etiology and progression to chronicity. Decreased innervation, with subsequent loss of skin sensitivity, prevents the patient to feel the skin lesions in a timely manner. Low vascularization also hinders adequate tissue oxygenation and nutrition, and diminishes accessibility of immune components and cells to the wounded areas, further impairing an adequate immune response (Forlee, 2011; Barshes et al., 2013; **Figure 1**).

Typically, a breach in the skin triggers a local cascade of inflammatory signals produced by immune cells (macrophages and dendritic cells, among others) in response to pathogen-associated molecular patterns (PAMPs) of foreign microbes or commensal skin microbiota that easily access the affected skin (Grice and Segre, 2012; Chu and Mazmanian, 2013). Increased levels of histamine and other vasodilators promote higher local blood flow and efflux of plasma to the site, which elicits swelling and heating responses, to prevent further microbial, access to the lesion. Complement and other plasma molecules, including antibodies, start clogging microbial cells in an attempt to control their growth and allow their subsequent phagocytosis and destruction (Chehoud et al., 2013). Other components like plasmin, platelets, and coagulants form a net of fibrinogenic tissue in the lesion to prevent blood release and further entrance of foreign components. This fibrotic tissue also functions as the scaffold for tissue regeneration (Winyard, 2003). However, recent evidence suggests that the skin immune response is not totally under control. Naik et al. (2012) reported that lipoteichoic acid (LTA) produced by staphylococci inhibits skin inflammation by a Toll-like receptor-dependent pathway. Another study demonstrated that complement component C5a receptor inhibition decreased the skin microbiota diversity, and that commensal microbiota was able to regulate the expression of some complement genes in the skin, thus regulating its immunity (Chehoud et al., 2013). Both studies highlight the direct microbial participation in the regulation of our skin immune response. This interplay between our immune system and our microbiota is now acknowledged to have important implications in the skin health–disease balance.

Apart from being exposed to the environment, our largest organ is completely covered with microbes, with an estimate of about 1 billion microbial cells per cm^2^ of skin covering its surface and extending down into the appendages and glands (Kong and Segre, 2012). Skin microbial communities protect from pathogens invasion, regulate local pH, and are able to respond rapidly to sudden environmental changes (Kong and Segre, 2012; Chu and Mazmanian, 2013; Foxman and Rosenthal, 2013). When a lesion occurs, skin microbiota gains access to the affected tissues, which may help triggering local immune responses by providing the necessary PAMPs signals for the inflammatory cascade to ignite (Ki and Rotstein, 2008). However, the skin microbiota composition may also ultimately determine if such interaction will have a beneficial or detrimental outcome to the host, especially if immune responses are impaired, as is the case of diabetic patients, thus at a higher risk of developing DFU.

Generally, the four dominant phyla of bacteria residing on the skin are the Actinobacteria, Proteobacteria, Firmicutes, and Bacteroidetes (Hannigan and Grice, 2013). Variability between individuals (inter-variability) and between different body sites (intra-variability) is a common trend (Human Microbiome Project Consortium [HMPC], 2012). However, the dominant genera of skin bacteria are relatively stable and include *Staphylococcus, Propionibacterium*, and *Corynebacterium*, and less frequently *Streptococcus* and *Pseudomonas*, which account for the observed interpersonal variability. However, the relative abundance of these dominant genera varies enormously according to the skin site and microenvironment (Costello et al., 2009; Grice et al., 2009; Nakatsuji et al., 2013). For instance, sebaceous, sweat, and follicle glands regions with a particular lipid and antimicrobial peptides’ (AMPs) content are characterized by specific microbial communities (Kong and Segre, 2012). Interestingly, bacteria, or at least bacterial genes, have also been identified in the subepidermal layers of the skin (Nakatsuji et al., 2013) reinforcing the close interaction of the skin microbiota with cells of the inner layers of the skin and their anticipated mutual regulation.

One study comparing the healthy skin microbiota of the foot and the forearm of 30 diabetic patients with 30 non-diabetic individuals showed statistically significant differences in the microbiota composition and diversity of the foot skin but not of the forearm (Redel et al., 2013). This study showed that the phylum Firmicutes is more prevalent in non-diabetic foot skin while the phylum Actinobacteria, more specifically the genus *Corynebacterium*, is more prevalent in diabetic foot skin, along with higher carriage rates of *Staphylococcus aureus* in the latter. Authors hypothesized that the characteristic low levels of eccrine sweat glands of the diabetic feet associated to lower levels of the AMP dermcidin which is active against *S. aureus*, could partly explain their observation, but they did not test that hypothesis. Also, since no follow up of the enrolled participants to determine DFU emergence was conducted, no extrapolation to possible DFU prognostic microbial signatures was attainable with this study. Other studies with similar goals are absent from the literature, as they focus exclusively on already existing and active chronic DFU microbiota (Dowd et al., 2008; Price et al., 2009; Grice et al., 2010; Gardner et al., 2013; Wolcott et al., 2016). Some studies compare DFU with contralateral intact skin (Gontcharova et al., 2010; Oates et al., 2012), but none is focused exclusively on diabetic foot skin prior to DFU emergence, which may indeed hold promising predictive, therefore preventive, potential.

Comparative studies, particularly between diabetic patients with and without clinical prognosis of DFU, would be helpful to unravel DFU prognostic microbial signatures. Apart from being potentially good diagnostic markers, useful for the implementation of directed antibiotherapy, these signatures could also allow the development of novel therapeutic strategies. Prebiotics, probiotics, or symbiotic formulations to modulate potentially pathogenic skin microbiota toward a novel microbiota composition useful to prevent skin lesions, similar to what is currently under investigation regarding regulation of the gut microbiota in the context of gut chronic inflammatory diseases (Ringel et al., 2012; Ford et al., 2014), could be a possibility.

## Progression of Diabetic Wounds to Chronic Stages and the Role of Microbes

**FIGURE 3:**
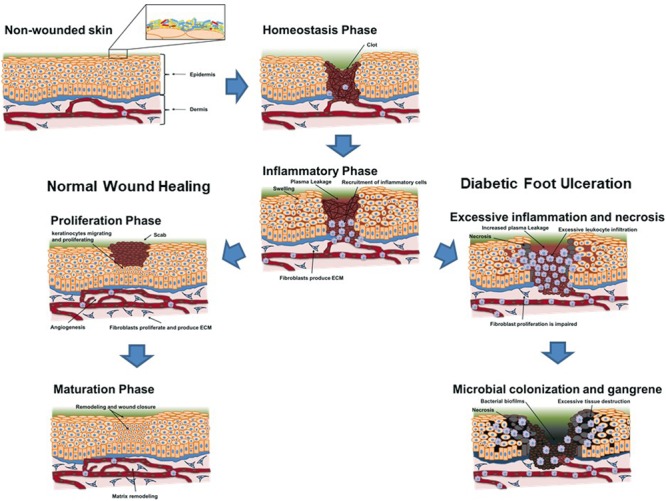
Schematic overview of the evolution of a skin lesion toward a regular remission and closure (left scheme – normal wound healing) and toward chronicity (right scheme – diabetic foot ulceration) in a diabetic patient. The influence of underlying conditions and microbial bioburden is briefly schematized to help visualize their role in wound evolution toward ulceration, as well as their non-impairing presence when regular closure of the wound occurs.

Wound healing is a complex and sequential process that involves blood coagulation and maintenance of hemostasis, immunological response against infecting pathogens and cell debris, re-epithelization of the damaged tissue, scar-tissue formation, and ultimately tissue remodeling (Moura et al., 2014). For wound healing to succeed, various processes in each phase need to occur in synchrony, so that the skin barrier is rapidly re-established and the infection is eliminated. However, in some diabetic patients, wound healing is impaired, with the different phases not occurring in synchrony, leading to chronic ulcers that fail to heal. The major changes that impair wound healing in DFUs are summarized in **Table 1** and **Figure 3**.

**Table 1:** Major changes observed in the different phases of wound healing in diabetic foot ulcers, when compared to non-diabetic skin lesions.

Phases of wound healing	Major changes observed in diabetic ulcers	References
Hemostasis	Hypercoagulable state with decreased fibrinolysis Denser fibrin clots and elevated cellular fibronectin	Erem et al., 2005; Konieczynska et al., 2017
Inflammatory	Excessive pro-inflammatory cytokine secretion Excessive M1 macrophage polarization Excessive tissue destruction Reduced blood flow to the wound Restriction of leukocyte chemotaxis	Moura et al., 2016; Kraakman et al., 2014; Hsu et al., 2014; Rogers et al., 2011; Gillitzer and Goebeler, 2001
Proliferation	Decreased keratinocyte proliferation and migration Decreased fibroblast ECM production Diminished angiogenesis Impaired endothelial progenitor cell mobilization	Lan et al., 2008; Law et al., 2012; Galiano et al., 2004; Liu and Velazquez, 2008
Maturation	Defective remodeling of ECM by fibroblasts Increased levels of host and bacterial MMPs promote ECM degradation	Maione et al., 2016; McCarty et al., 2012

Diabetic foot ulcers are usually painless, due to lower sensor neuron concentration, which leads to decreased pressure, heat, and pain sensation (Turns, 2011). Besides neuropathy and the biochemical imbalance, diabetic patients are usually in a hypercoagulable state with decreased fibrinolysis (Erem et al., 2005), leading to the formation of denser fibrin clots upon vascular injury (Konieczynska et al., 2017). Angiogenesis is also usually impaired, leading to a decreased blood flow to the wound site (Rogers et al., 2011), impairing inflammation through the restriction of leukocyte chemotaxis and, consequently, impairing pathogen elimination (Gillitzer and Goebeler, 2001). Excessive pro-inflammatory cytokine secretion (Moura et al., 2016), specially TNF-alpha (Xu et al., 2013) and excessive M1 macrophage polarization (Kraakman et al., 2014) lead to increased tissue destruction (Hsu et al., 2014), prolonging the healing time, and potentiating pathogen colonization and biofilm formation (Rahim et al., 2017). Byproducts of the exacerbated immune response, such as granzymes and perforins, promote extracellular matrix (ECM) degradation and impair keratinocyte migration through the cleavage of epidermal growth factor receptor, hindering tissue re-epithelization (Merkulova et al., 2016) and prolonging wound healing time, rendering it chronic.

The diminished angiogenesis has a large impact on wound healing, as demonstrated by the accelerated healing observed in patients when treated topically with vascular endothelial growth factor (VEGF) (Galiano et al., 2004). Immune cells also seem to be important for lymphatic vessel formation through the production of VEGF (Maruyama et al., 2007). Reduced blood flow also decreases non-immune cell migration, namely keratinocytes, fibroblasts, and endothelium progenitor cells, responsible for tissue regeneration (Liu and Velazquez, 2008). Hyperglycemia decreases keratinocyte proliferation and migration (Lan et al., 2008), thus diabetic conditions further impair fibroblast ECM production (Law et al., 2012), which favors bacterial wound colonization. ECM remodeling is also significantly impaired, either due to altered ECM deposition (Maione et al., 2016) or due to ECM degradation by metalloproteinases secreted by the wound cells or by infecting bacteria (McCarty et al., 2012).

Apart from vascular and neuropathies, inflammation is the most significant factor in wound chronicity, with higher degrees of inflammation relating to poorer outcomes in wound response to therapy (Ming et al., 2012; Chehoud et al., 2013; Mirza et al., 2014; Moura et al., 2014; Leal et al., 2015). Acute inflammation is considered the primary, innate response to foreign cells in a site, the majority against microbial cells, and can last a few days (Ki and Rotstein, 2008). Yet, if the causative agent persists in the wound site, acute inflammation can progress to chronic inflammation, characterized by constantly high levels of macrophages and other inflammatory components. High production of toxic byproducts, particularly reactive oxygen species, affects not only the invading microbes but also the wound tissues (Ming et al., 2012). This leads to increased and progressive tissue degradation, greatly impairing wound healing (Mirza et al., 2014; Moura et al., 2014; Leal et al., 2015) exacerbating the impairment already caused by the infection (Grice et al., 2010; Grice and Segre, 2012; Gardner et al., 2013; Wolcott et al., 2016). Inflammation is also modulated by the release of neuropeptides from skin nerve endings. In fact, neuropeptides, like substance P, neuropeptide Y, and neurotensin, regulate the immune function of skin cells, like the degranulation of mast cells and the release of pro- or anti-inflammatory cytokines and chemokines (da Silva et al., 2010). They also regulate vasodilation and vasoconstriction and the proliferation and differentiation of various cells (Roosterman et al., 2006). However, it is relevant to highlight that some of these inflammation factors can be specifically controlled by our skin microbiota apart from the intrinsic regulation by the immune system. In fact, after a skin lesion, the inflammatory response can be dampened by staphylococci produced LTA linked to a Toll-like receptor-2 mechanism (Lai et al., 2009). LTA is also important for mast cells maturation, that remain immature in the skin of germ-free mice (Wang et al., 2017). Cytokines levels and production in the skin were also demonstrated to be influenced by the skin microbiota (Naik et al., 2012). Blockage of complement signaling, via a C5a receptor, decreased skin microbiota composition and diversity, which induces itself the expression of those complement genes (Oh et al., 2013). Moreover, some skin microbial commensals can themselves have antimicrobial properties that decrease skin colonization with pathogens, as observed in a study regarding atopic dermatitis and the role of coagulase-negative staphylococci against *S. aureus* (Nakatsuji et al., 2017). A study on acne also demonstrated this protective property of the commensal microbiota, highlighting the importance of the balance of commensals in protection against disease and not just their mere presence in the skin (Barnard et al., 2016).

Some literature distinguishes wound colonization from wound infection, the latter occurring only when a specific level of pathogenic microbial load, typically >10^5^ colony forming units (CFUs) per gram of tissue is reached (Spichler et al., 2015). So, the term “critical colonization” is frequently used to define the threshold that determines the onset of an infection, i.e., pernicious effects for the host due to the presence and activity of pathogenic microorganisms. However, this quantitation is supported only in culture-based methods which is a quite narrow approach, as they fail to detect 99% of the colonizing bacteria (Grice et al., 2010; Grice and Segre, 2012; Kong and Segre, 2012), including fastidious, slowly growing, and anaerobic bacteria, already described as prevalent in DFU (Dowd et al., 2008; Price et al., 2009; Smith et al., 2016; Wolcott et al., 2016). Also the expression “microbial colonization” may give a misleading idea by suggesting that microbial colonizers are innocuous to the wound, which may not be the case. Not only low levels of specific microorganisms can cause greater negative impact than high burden of others less virulent but, more importantly, microbial consortia frequently display pathogenic traits not observed individually, as a consequence of interspecies quorum-sensing or quorum-quenching phenomena. This is highly relevant in DFU environment, where consortia of polymicrobial non-cultivable biofilms prevail (Dowd et al., 2008; Santos et al., 2016; Wolcott et al., 2016).

Although it is well established that many of the species found in chronic wounds are commensals in the healthy skin, there are clear differences between DFU and healthy skin microbiota in terms of composition and diversity (Grice and Segre, 2012). A study comparing 23 paired samples of DFU microbiota with intact foot skin showed the latter to have higher bacterial diversity than the wound, both at the genus and species levels, and also less prevalence of opportunistic pathogens (Gontcharova et al., 2010). Increased biological diversity stabilizes an ecosystem against biological and environmental changes (Cleland, 2011), which may explain the lower incidence of pathogens in healthy skin compared to that of DFU. Lower microbial diversity has been associated with other dysbiotic skin chronic disorders, like atopic dermatitis (Flores et al., 2014) and psoriasis (Alekseyenko et al., 2013), concurring with low microbial diversity favoring disease. Using different techniques, Oates et al. (2012) performed a similar study, including culture-based approaches. They showed that cultivable pathogens were exclusive to DFU but did not affect the wound bacterial load, diversity, or composition, as no distinct qualitative sequencing profiles were observed in the “infected” wounds comparatively to the “uninfected” (Oates et al., 2012). Another study, focused only in neuropathic DFU microbiota, observed higher bacterial diversity associated to deeper and more prolonged DFU, with higher levels of anaerobes and Proteobacteria particularly in the deeper wound layers, while the wounds that were more superficial and with shorter duration were associated with lower bacterial diversity and higher relative abundances of *Staphylococcus*, the majority identified as *S. aureus* (Gardner et al., 2013). This concurs with the time-dependent ecological stability of the wound and corresponding shifts in microbial loads and composition. A time-dependent study of wound microbiota evolution in diabetic and non-diabetic murine models showed not only statistically significant differences in microbial wound load over time between diabetic and non-diabetic wounds, with emphasis on Firmicutes species including *Staphylococcus*, but also in the local immune responses, demonstrating the intertwined combination of microbial and immunological factors in the skin’s response to injury (Grice et al., 2010). Smith et al. (2016) reinforced the role of anaerobes in DFU maintenance and demonstrated that the duration of ulceration appears to be more relevant to its microbiota composition and subsequent treatment approaches, than other aspects like new vs. recurrent ulcers.

Different sequencing methods and bioinformatics approaches often lead to different results, not related to real differences in the tested individuals (Human Microbiome Project Consortium [HMPC], 2012). Small cohort studies lack statistical robustness (Garg et al., 2008) to produce information that can be used worldwide, with the need for frequent geographic dependent epidemiology studies to confirm results and adjust them to local realities. A recent project on chronic wound microbiota addressed both problems by performing a study in 2963 patients with different chronic wounds (910 DFU, 916 venous ulcers, 767 decubitus ulcers, and 370 non-healing surgical wounds), using the same analytical methodology. Results suggested no demographic or wound type influence on the microbiota results, and demonstrated the prevalence of *Staphylococcus, Pseudomonas*, and anaerobes biofilms in the wounds (Wolcott et al., 2016). Apparently contradicting all the above mentioned literature that clearly demonstrates significant differences in immune cells and metabolites of the diabetic skin and wounds when compared to non-diabetics, what this study points out is that the findings obtained in diabetic wounds studies, particularly those related to novel treatment strategies, may be equally successful in other chronic wounds, as those present the same microbiota profile (and probably the same local immunological profile). With few, if any, ongoing studies for these types of severe skin lesions, these results may be highly relevant for future approaches to treat those ulcers.

Another two microbial aspects that have to be taken in consideration in DFU progression is the antibiotic resistance and biofilm profile of DFU microbiota, paramount for the success of DFU management.

## Chronicity Maintenance in Diabetic Wounds By Impaired Immune Response and Installed Microbiota

Diabetes and its associated complications have a long-term effect on the immune response, with a direct impact on wound healing (Acosta et al., 2008). Long-term hyperglycemia promotes the activation of NF-kB transcription factor (Dickinson et al., 2008), leading to a chronic inflammatory state (Acosta et al., 2008). The reduced blood flow restricts macrophage and T-cell chemotaxis (Galkowska et al., 2005) and keratinocyte, fibroblast, and endothelium progenitor cell migration to the wound (Liu and Velazquez, 2008). It also has a dramatic impact in the healing process, reducing oxygen availability and consequently ATP production (Rogers et al., 2011). In fact, hyperbaric oxygen therapy reliefs the need for lactic acid formation and improves wound healing (Jensen et al., 1990), but it does not cure DFU (Andrade and Santos, 2016). This is partially because increased mitochondrial activity leads to oxidative stress, which can offset the healing effect in the long term (Ma et al., 2013), but also because the lack of energy availability is not the only cause for the impaired immune function (Blakytny and Jude, 2009). Some models also propose that impaired wound healing in DFU results from a concomitant excess of pro-inflammatory cytokines like TNF-alpha, and deficit of anti-inflammatory and healing associated cytokines, like IL-10 and TGF-beta (Mi et al., 2007). This cytokine unbalance explains the excessive M1 macrophage polarization, accumulation of CD8^+^ T-cells, and excessive granzyme secretion (Kraakman et al., 2014) on the wound of diabetic individuals, but fails to explain why some DFUs surprisingly become chronic with uncontrolled infections.

Infections that cause a strong T-cell stimulation may cause a deleterious effect on T-cell receptor (TCR) repertoire diversity, thus causing a huge impact on the immune response to subsequent infections by different pathogens (Brunner et al., 2011). This effect is mainly due to the competition for resources (such as IL-2) that mediates the survival of naïve cells and limits the total T-cell count (Stockinger et al., 2004). Moreover, other studies also demonstrated that regular pathogen encounters have a significant negative effect on TCR repertoire diversity in aged individuals (Qi et al., 2014) and overall immune fitness, a phenomenon that is now called inflammaging (Franceschi et al., 2017). Diabetes leads to a significant reduction in TCR diversity and promotes accumulation of effector T-cells, which secrete large quantities of inflammatory cytokines, such as TNF-alpha and IFN-gamma further fueling chronic inflammation (Moura et al., 2016). Nevertheless, it has been demonstrated that there is a fair amount of plasticity in TCR repertoire, mainly due to cross-reactivity, that compensates for the reduction in TCR repertoire diversity (Armstrong et al., 2008), but this effect may not fully compensate the decreased diversity, thus limiting T-cells ability to defend us from endogenous pathogens, allowing bacterial colonization and biofilm formation in wounds (**Figure 3**).

Polymicrobial biofilms emerged recently as a tickpoint for wound chronicity (HØiby et al., 2015; Santos et al., 2016). Contrarily to planktonic microorganisms, typically associated to acute infections and usually easy to treat with low concentrations of appropriate antibiotics, biofilms are very difficult to treat and eradicate, requiring high doses of antibiotics that frequently fail, allowing the biofilm infection to become chronic and frequently fatal (Dowd et al., 2008; HØiby et al., 2015; Santos et al., 2016). Embedded in a self-produced extracellular polymeric matrix, microbial cells in a biofilm are protected not only from antibiotics but also from immune cells, with studies describing a low permeability of antibodies and neutrophiles in microbial biofilm matrices (Wolcott et al., 2016). Moreover, biofilm cells induce inflammation to promote local plasma leakage, which they use for nutrition, and host cells senescence by means of cytoskeleton rearrangement and mitosis or apoptosis inhibition (Wolcott et al., 2016), according to the biofilm needs. Another relevant aspect of polymicrobial biofilms is their ability to adapt and even thrive in adverse circumstances, due to the enhanced metabolic cooperation and gene expression regulation between the different sessile microbial cells there present (Dickschat, 2010; Wolcott et al., 2016). In general, increased biofilm diversity promotes its survival, resulting in more severe infections, also more difficult to treat (Dowd et al., 2008; Wolcott et al., 2016). All studies that consider biofilms in DFU indicate *S. aureus* and diverse anaerobes as its major components, and some also report *Pseudomonas aeruginosa* as prevalent (Grice and Segre, 2012; Wolcott et al., 2016).

Antibiotic resistance is another important aspect that greatly impacts DFU chronicity. Not only biofilm matrices prevent antibiotics to reach the embedded cells, but when they do they are at sub-optimal concentrations, significantly favoring the emergence of drug resistance (Hoffman et al., 2005; Dowd et al., 2008; Dickschat, 2010; Kalan et al., 2016; Santos et al., 2016; Strugeon et al., 2016). Moreover, some antibiotics may in fact favor biofilm formation. Also, highly concerning is the fact that the majority of DFU cultivable pathogens exhibit multidrug-resistance profiles (Lipsky et al., 2012; Banu et al., 2015; Santos et al., 2016) and the most prevalent colonizers are known multidrug-resistant pathogens. *S. aureus* and *P. aeruginosa* are the first and fifth most prevalent pathogens worldwide and the most difficult to treat due to their high ability to develop multidrug resistance (Centers for Disease Control and Prevention, 2013; World Health Organization [WHO], 2017). Biofilm architecture and activity, as well as its relevance to infections only started to be appreciated and understood by the end of 20th century, while the development of the currently available antibiotics occurred a few decades earlier, thus exclusively directed to planktonic microorganisms (Santos et al., 2016). Therefore, it is not surprising to observe the low efficiency of antibiotherapy in DFU management (Lipsky et al., 2012). In addition to all this, it is also important to highlight that the International Working Group on Diabetic Foot (IWGDF), that emanate worldwide recommendations for DFU management and treatment, considering that there are not enough evidences to support the long-term use of local antibiotherapy (Lipsky et al., 2015), advocate the use of systemic antibiotics in DFU treatment instead of topical ones, regardless the fact that low vascularization may prevent them from reaching the wound and, if they do, the sub-optimal concentration at which they will reach the wound site will favor drug-resistance acquisition ability, as already demonstrated at a genetic level (Strugeon et al., 2016).

This combination of diverse multidrug-resistance pathogens stably embedded in polymicrobial biofilms and an antibiotic treatment regimen that prevents adequate concentration in the wound site gives a poor outcome to DFU antibiotherapy that needs to be rapidly addressed in order to change the current paradigm of DFU management.

## Treatment Strategies and Future Perspectives for Diabetic Chronic Wounds

Chronic wounds do not heal naturally and some degree of therapeutic intervention is always required. Following the diagnosis of the wound chronicity stage, corresponding clinical treatments are initiated. Debridement of compromised tissues, use of specialized dressings, and administration of broad and sometimes narrow spectrum of antibiotics are the most frequently used approaches to treat diabetic chronic wounds (Lipsky et al., 2015). However, success rates are still far from being satisfactory, with less than half of the patients responding positively to this approach, and hospitalization and amputation often required, although with poor outcomes for those patients (Forlee, 2011; Icks et al., 2011; Kerr, 2012; Barshes et al., 2013; World Health Organization [WHO], 2016; **Figure 2**). Although it is demonstrated that antibiotic administration can, in several cases, further impair wound healing progression (Dowd et al., 2008; Lipsky et al., 2012; Banu et al., 2015; Santos et al., 2016), without alternative options physicians continue to administer systemic antibiotics (Lipsky et al., 2015), in the hope that something changes in the global health condition of the patient that allows the wound to enter a remission stage.

Finding new alternatives for the treatment of this life-threatening problem is urgent. Several efforts are being made with that goal in mind, with reports suggesting the positive intervention of neuropeptides, microRNAs, and redox control in wound healing (Banerjee and Sen, 2013; Dhall et al., 2014; Moura et al., 2014; Leal et al., 2015), among others, bringing thus the hope of new roads to explore in the quest for future new therapeutic strategies. Recently, several studies on microbial biofilms architecture and microbiome analysis allowed an increasing awareness of the important role microbes have in wound chronicity and studies focused on controlling wound biofilm microbiota are now rapidly emerging.

Walker and colleagues as well as Metcalf and colleagues have developed and successfully tested *in vitro* and *in vivo* next-generation antimicrobial wound dressings specifically directed to the disruption of microbial biofilms, with very promising results (Said et al., 2014; Walker et al., 2014; Metcalf et al., 2017). AMPs and their synthetic derivatives have also recently emerged as potential alternatives to kill pathogens in the wound site, without promoting drug-resistance phenomena. As AMPs are naturally secreted by the host as part of the immune response to PAMPs, and thus cohabite and evolve with our commensal microbes, it is not expected that their therapeutic use will promote drug resistance (Santos et al., 2016). Bacteriophages are also an emerging alternative that relies on the fact that bacteriophages are bacteria dependent, thus posing no harm to human cells, in addition to being fully target-directed (Santos et al., 2016). However, bacteriophages are not expected to have activity against eukaryotic microbes, whose relevance in chronic wounds progression has also recently been acknowledged (Kalan et al., 2016), rendering this alternative a relevant limitation.

Application of the acquired knowledge on the wound microbiota composition can also be used to devise innovative therapeutic approaches, using the concept of probiotics (microbes with specific functions) or prebiotics (nutrients to enhance existing microbial functions), to reverse gut chronic microbiota associated diseases to a status of non-chronic, non-impairing, condition (Ringel et al., 2012; Ford et al., 2014). Fecal transplants are becoming increasingly accepted in the clinic to treat *Clostridium difficile* intestinal infections, with excellent results (Gough et al., 2011), so similar approaches to other debilitating infections can become a reality in the near future, including DFU. In fact, evidences of microbiota transplants in the skin are already being tested for atopic dermatitis (Myles et al., 2016; Nakatsuji et al., 2017). Phage therapy, used to modulate skin microbiota, is also being considered as an option, considering the higher carriage of phage genes in healthy individuals when compared to acne patients (Barnard et al., 2016).

Other more empirical approaches are also being tested for wound healing with positive results, including the application of natural products like honey (Henriques et al., 2006), essential oils (Woollard et al., 2007), and even maggot debridement (Sherman, 2014). Altogether, these studies open the possibility of diverse new roads to explore in the quest for future new and more efficient therapeutic strategies to treat chronic wounds in diabetic and non-diabetic patients. The knowledge emerging from these studies can be used to optimize, refine, and combine compounds and strategies with positive results in wound management.

However, it is important not to forget that chronic wounds are a multifactorial condition, depending on the usually impaired patient’s healing mechanisms, on the wound’s microbial colonizers of diverse origins (bacterial, fungal, viral), composition, presentations (planktonic, biofilms), and their genetic arsenal (drug resistance genes, virulence factors production, and regulatory mechanisms) and also on the environmental determinants that surround the wound (moisture, temperature, pH, among others), all intertwined with each other and with triggering determinants (**Figure 1**). Strategies to solve this extensively impairing health-problem will require focus on all aspects and not just in one, as done in the past. A multidisciplinary, holistic, approach is of paramount importance to address this complex health issue (at a personal, social, and economic level), and most certainly others.

## Author Contributions

SP designed the review structure and goals, wrote the microbiology, part of the immunology and the interdisciplinary texts, coordinated the input of other authors, made and approved the final revision prior to submission. JM wrote the immunology text, generated the figures and tables, and approved the final version of the manuscript. EC revised the text regarding immunology and approved the final version of the manuscript. NE accompanied the writing, discussed ideas, revised the text, and approved the final version of the manuscript.

## Conflict of Interest Statement

The authors declare that the research was conducted in the absence of any commercial or financial relationships that could be construed as a potential conflict of interest.
